# European Society of Cardiology and Radical Health Festival Helsinki join forces to transform healthcare as we know it

**DOI:** 10.1093/ehjdh/ztad036

**Published:** 2023-06-02

**Authors:** Gerhard Hindricks

**Affiliations:** German Heart Center of the Charite, Berlin, Germany


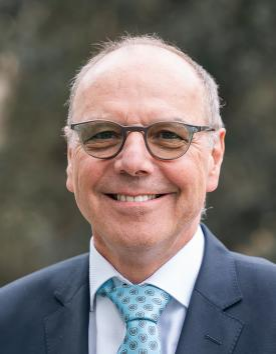


## This is our wake-up call!

### Intro

Earlier this year, the European Society of Cardiology (ESC) announced its partnership with Radical Health Festival Helsinki. This new pan-European event is driven by a genuine ambition to transform healthcare, deliver value, improve clinical and financial outcomes, and make health systems sustainable. Taking place this summer from 12 to 14 June, it gathers partners from across the European digital health ecosystem, who believe only radical change can make that happen.

The ESC is the festival’s lead clinical partner. Prof. Gerhard Hendricks, FESC, Chair of the ESC’s Digital Health Committee, explains why the ESC is joining forces with the festival, what its contributions will be, and how this will support the ESC’s long-terms goals for digital health.

Prof. Gerhard Hindricks, FESC

## Why this collaboration?

Digital tools, artificial intelligence (AI), and big data analytics play a prominent role in medicine and science. Digital health is transforming the way we collect patient information and conduct research; it is revolutionizing how we diagnose, monitor, and deliver healthcare to our patients; it is disrupting the traditional role of healthcare professionals and profoundly impacting the relationship between patients, physicians, and other healthcare providers.

As a pioneer and provider of education, recommendations, and resources for the global cardiology community, the ESC has long held digital health as a strategic priority. This important area is covered by dedicated tracks at our congresses, webinars, and roundtables and this journal, which has been the mouthpiece of the ESC since 2020.


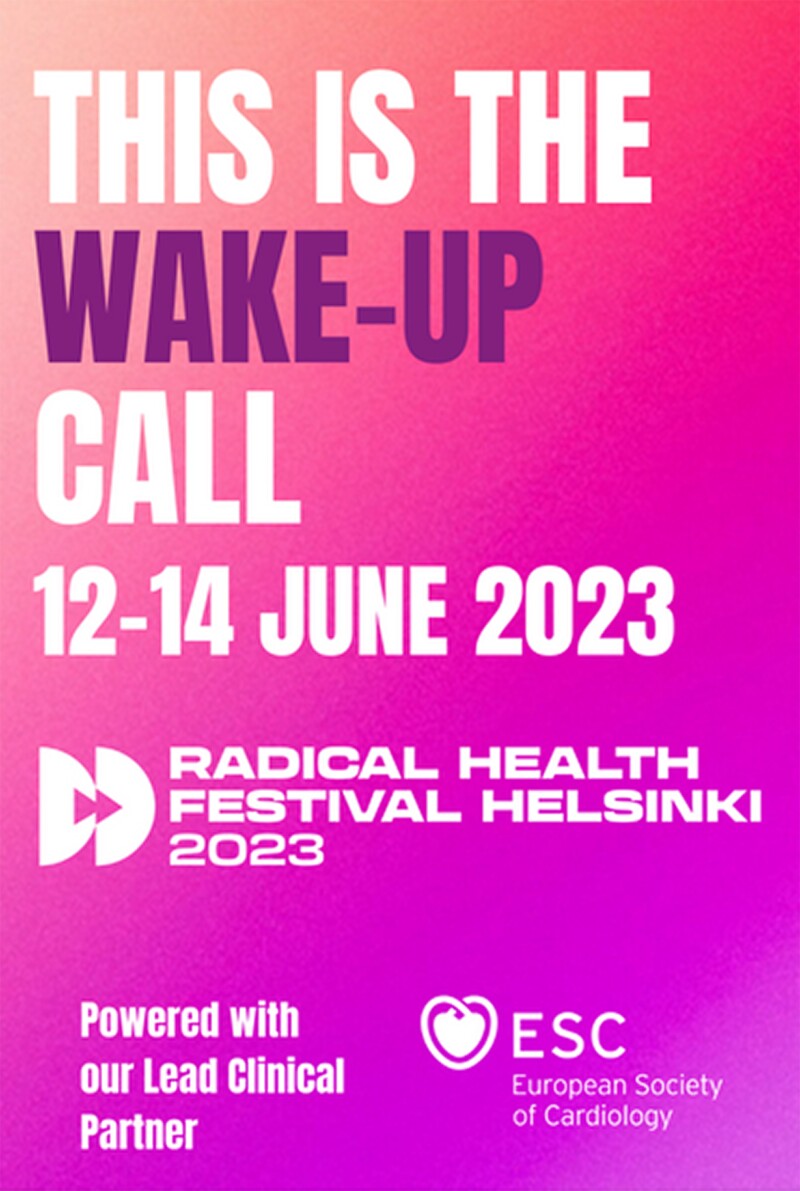


However, clinicians and scientists are not the only actors in a digital health revolution—a revolution that the entire world is trying to embrace, regulate, optimize, and pay for. Radical Health Festival Helsinki will gather the entire European health innovation ecosystem under one roof. This includes policy-making authorities and public health agencies; patient organizations, hospitals, and health systems; pharma and medtech groups; public and private insurers; major information technology (IT) vendors; and startups, entrepreneurs, and investors.

As Dr Päivi Sillanaukee, Ambassador for Health, Ministry for Foreign Affairs, Finland, puts it, ‘*The best way to boost change, implement innovations and share best practices is to bring together people with ideas, knowhow and a powerful mission*.’

Collaborating with partners like Radical Health Festival Helsinki puts the ESC at the heart of high-level discussions and enables it to maintain and develop a very different type of network to what medical societies may have had in the past. But this will ensure that the ESC stays abreast of what is happening in digital health to support its members, as well as keeping the interests and concerns of the cardiovascular community and our patients, at heart of the digital health revolution.

## The European Society of Cardiology’s role

As the festival’s lead clinical partner, the ESC will offer a two-day track covering key topics from digital health innovations vs. regulations (MDR/AI-Act) and connected health (mHealth and wearables/patient monitoring), to digital therapeutics, *in silico* trials (diagnostics and regulatory), and patient empowerment. Access full details of the ESC digital health track at https://radicalhealthfestival.messukeskus.com/esc-rhfh/.

Sessions will be multi-stakeholder roundtables discussing how these topics impact not only cardiovascular medicine, but also those involved in creating it, regulating it, applying it, and paying for it as well as how to ensure patients understand and interact with it.

To complement these sessions, the ESC stand will be there to explain who the ESC is, what we already do in the digital health space, and where we are seeking collaboration to be successful in our future goals.

## Long-terms goals

This latest collaboration is part of a journey the ESC has embarked upon through its 5-year Strategic Plan. The plan has identified six major developments and trends (‘future scenarios’) that will influence the ESC and the medical and scientific environment. Digital health is one of these.

A number of actions are highlighted in the strategic plan for digital health, including

- partnering with a data specialist to be a European driver for data definition in the digital health area in cardiovascular medicine
- setting standards and providing training to cardiovascular health professionals in digital health technologies

Such far-reaching objectives can only be achieved by working hand in hand with all those within the digital health ecosystem, and events like Radical Health Festival Helsinki are exciting opportunities for the ESC and all those who want to shape the future of cardiology.

Find out more information about Radical Health Festival Helsinki at https://radicalhealthfestival.messukeskus.com/.

For more information about ESC’s strategic plan, visit escardio.org/strategic-plan: https://escardio.org/The-ESC/What-we-do/strategic-plan.

## Data Availability

No new data were generated or analysed in support of this research.

